# Supplementary material for: Heat adaptation of phage T7 under an extended genetic code
Source: Virus Evol. 2021 Dec 1;7(2):veab100. doi: 10.1093/ve/veab100 (PMC8923235; doi:10.1093/ve/veab100)
Supplement: veab100_Supp [file veab100_supp.zip › Sequencing_Analysis.zip!/Sequencing_Analysis/A1/evidence/DEL_15.html]

BRESEQ :: Evidence


| Predicted mutation | | | | | | |
| --- | --- | --- | --- | --- | --- | --- |
| evidence | seq id | position | mutation | annotation | gene | description |
| RA | NC\_001604.1 | 37,030 | Δ2 bp | intergenic (–/–) | – / – | –/– |

| Read alignment evidence... | | | | | | | | | | | |
| --- | --- | --- | --- | --- | --- | --- | --- | --- | --- | --- | --- |
|  | seq id | position | | ref | new | freq | score (cons/poly) | reads | annotation | genes | product |
| \* | NC\_001604.1 | 37,027 | 0 | A | . | 80.0% | 7432.5 / inf | 3245 | intergenic (–/–) | *–/–* | –/– |
| Reads supporting (aligned to +/- strand):  **ref** base A (324/323);  **new** base . (1367/1227);  **total** (1691/1554) | | | | | | | | | | | |
| Fisher's exact test for biased strand distribution *p*-value = 2.35e-01 | | | | | | | | | | | |
| Kolmogorov-Smirnov test that lower quality scores support variant *p*-value = 1.00e+00 | | | | | | | | | | | |
| \* | NC\_001604.1 | 37,028 | 0 | C | . | 81.8% | 7606.0 / inf | 3245 | intergenic (–/–) | *–/–* | –/– |
| Reads supporting (aligned to +/- strand):  **ref** base C (314/273);  **new** base . (1370/1264);  **total** (1691/1554) | | | | | | | | | | | |
| Fisher's exact test for biased strand distribution *p*-value = 5.23e-01 | | | | | | | | | | | |
| Kolmogorov-Smirnov test that lower quality scores support variant *p*-value = 1.00e+00 | | | | | | | | | | | |

Only 100 of 3408 total aligned reads displayed.  

`CACATCTTAGGTGGCCTTGCTGGTGCTCTTGAAGAGTACAAAGAGAAAGTCGGTGATAACGGTCTTACGGATGATGATATTTACACATTACAGTGATATACTCAAGGCCACTACAGATAGTGGTCTTTATGGATGTCATTGTCTATACGAGATGCTCCTACGTGAAATCTGAAAGTTAACGGGAGGCATTATGCTAGAATTTTTACGTAAGCTAATCCCTTGGGTTCTCGCTGGGATGCTATTCGGGTTAGGATGGCATCTAGGGTCAGACTCAATGGACGCTAAATGGAAACAGGAGGTACACAATGAGTACGTTAAGAGAGTTGAGGCTGCGAAGAGCACTCAAAGAGCAATCGATGCGGTATCTGCTAAGTATCAAGAAGACCTTGCCGCGCTGGAAGGGAGCACTGATAGGATTATTTCTGATTTGCGTAGCGACAATAAGCGGTTGCGCGTCAGAGTCAAAACTACCGGAACCTCCGATGGTCAGTGTGGATTCGAGCCTGATGGTCGAGCCGAACTTGACGACCGAGATGCTAAACGTATTCTCGCAGTGACCCAGAAGGGTGACGCATGGATTCGTGCGTTACAGGATACTATTC  >  NC_001604.1/36727‑37328`  

                                                                                                                                                                                                                                                                                                            `||`                                                                                                                                                                                                                                                                                                              
   `ctCTTAGGTGGCCTTGCTGGTGCTCTTGAAGAGTAAAAAGAGAAATTCGGTGATAACTGTTTTACGGATGATTATATTTACATATTACAGTGATATACTCAAGTCCACTACAGATAGTGGTCTTTATGGATGTCATTGTCTATACGAGATGCTCCTACGTGAAATCTGAAAGTTAACGGGAGGCATTATGCTAGAATTTTTACGTAAGCTAATCCCTTGGGTTCTCGCTGGGATGCTATTCGGGTTAGGATGGCATCTAGGGTCAGACTCAATGGACGCTAAATGGAAACAGGAGGTacaa                                                                                                                                                                                                                                                                                                            <  1:473889/300‑2 (MQ=255)`  
   `aTCTTAGGTGGCCTTGCTGGTGCTCTTGAAGAGTAAAAAGAGAAAGTCGGTGATAACGGTCTTACGGATGATGATATTTATACATTACAGTGATATACTAAAGGCCACTACAGATAGTGGTCTTTATGGATGTCATTGTCTATACGAGATGCTCCTACGTGAAATCTGAAAGTTAACGGGAGGCATTATGCTAGAATTTTTACGTAAGCTAATCCCTTGGGTTCTCGCTGGGATGCTATTCGGGTTAGGATGGCATCTAGGGTCAGACTCAATGGACGCTAAATGGAAACAGGAGGTacaa                                                                                                                                                                                                                                                                                                            <  1:350271/301‑2 (MQ=255)`  
   `aTCTTAGGGGGCCTTGCTGGTGCTATTGAAGAGTACAAAGAGAAAGTCGGTGATAACGGTCTTACGGATGATGATATTTACACATGACAGTGATATACTCAAGGCCACTACAGATAGTGGTCTTTATGGATGTCATTGTCTATACGAGATGCTCCTACGTGAAATCTGAAAGTTAACGGGAGGCAGTATGCTAGAATTTTTCCGTAAGCTAATCCCTTGGGGTCTCGCTGGGATGCTATTCGGGTTAGGATGGCATCTAGGGTCAGACTCAATGGACGCTAAATGGAAACAGGAGGTacaa                                                                                                                                                                                                                                                                                                            <  1:202450/301‑2 (MQ=255)`  
     `ttttggTGTCCTTGCTGTTGCTATTGAAGCTTACAAAGGTAAAGTCGGTGATAACAGTATTCTCGATCATGATATTTACCCATTACAGTTATAGACTCAAGGTCACTACAGATAGTGGTCATTATTGATGTCATTGTCTATACGAGATGCTCCTACGTGAAATCTGAAAGTTAACGGGAGGCATTATGCTAGAATTTTTACGTAAGCTAATCCCTTGGGTTCTCGCTGGGATGCTATTCGGGTTAGGATGGCATCTAGGGTCAGACTCAATGGACGCTAAATGGAAACAGGAGGTacaatg                                                                                                                                                                                                                                                                                                          <  1:431474/297‑4 (MQ=255)`  
     `cTTAGGTGTCCTTGCTGGTGCTCTTGAAGAGTACAAAGAGAAAGTCGGTGATAACGGTCTTACGGATGATGATATTTACACATTACAGTGATATACTCAAGGCCACTACAGATAGTGGTCTTTATGGATGTCATTGTCTATACGAGATGCTCCTACGTGAAATCTGAAAGTTAACGGGAGGCATTATGCTAGAATTTTTACGTAAGCTAATCCCTTGGGTTCTCGCTGGGATGCTATTCGAGTTAGGATGGCATCTAGGGTCAGACTCAATGGACGCTAAATGGAAACAGGAGGTacaatg                                                                                                                                                                                                                                                                                                          <  1:179685/301‑4 (MQ=255)`  
     `cTTAGGTGGCCTTGCTGGTGCGCTTGAAGAGTACAAAGAGAAAGTCGGAGATAACGGTCATACGGATGATGATATTTAAACATTACAGTGATATACTCAAGGCCACTACAGATAGTGGTCTTTATGGATGTCATTGACTATACGAGATGCTACTACGTGAAATATGAAAGTTAACGGGAGGAATTATGCTAGAATTTTTACGTAAGCTAATCACTAGGGTTCACGTTGGGATGCTATTCGGGTTAGGATGGCAACTAGGGAAAGATACAATGGAAGAAAAAGGGAAACAGGAGGTACACaa                                                                                                                                                                                                                                                                                                          >  1:306460/1‑301 (MQ=255)`  
     `agctacaggCCTTGCTGGTGCTCTTGAAGAGTACAAAGAGAAAGTCGGTGATAACGGTCTTACGGATGATGATATTTACACATTACAGTGATATACTCAAGGCAACTACAGATAGTGGTCTTTATGGATGTCATTGTCTATACGAGATGCTCCTACGTGAAATCTGAAAGTTAACGGGAGGCATTATGCTAGAATTTTTACGTAAGCTAATCCCTTGGGTTCTCGCTGGGATGCTATTCGGGTTAGGATGGCATCTAGGGTCAGACTCAATGGACGCTAAATGGAAACAGGAGGTacaatg                                                                                                                                                                                                                                                                                                          <  1:210652/294‑4 (MQ=255)`  
      `ttAGGTGGCCTTGCTGGTGCTCTTGAAGAGTACAAAGAGAAAGTCGGTGATAACGGTCTTACGGATGATGATATTTACACATTACAGTGATATACGCAAGGCCACTACAGATAGTGGTCTTTATGGATGTCATTGTCTATACGAGATGCTCCTACGTGAAATCTGAAAGTTAACGGGAGGCATTATGCTAGAATTTTTACGTAAGCTAATCCCTTGGGTTCTCGCTGGGATGCTATTCGGGTTAGGATGGCATCTAGGGTCAGACTCAATGGACGCTAAATGGAAACAGGAGGTACACAAt                                                                                                                                                                                                                                                                                                         <  1:464806/301‑1 (MQ=255)`  
          `agggCATTTCTGGTGCGCTTTAAGAGGACAAGAAGAAAGTGGGTGTAAACTGTGTTACGGATGATGATATTTACACTTTACAGTGATATACTCAAGGCCATTACGAATAGTGGTTTTTATGGATGTCATTGTCTATACGAGATGCTCCTACGTGAAGTCTGAAAGTTAACGGGAGGAATTATGCTAGAATTTTTACGTAAGCTAATCCCTTGGGTTCTCGCT‑GGATGCTATTCGGGTTAGGATGGCATCTAGGGTCAGACTCAATGGACGCTAAATGGAAACAGGAGGTACACAATGAGTa                                                                                                                                                                                                                                                                                                    <  1:255048/299‑1 (MQ=255)`  
                `ttGGTGGTGCTCTTGAAGAGTAAAAATGGAAAGTTGGTGATAACGGTCTTACGGATGATGATATTTACACATTACAGTGAAATACTCAAGGCCACTAAAGATAGTGGTCTTTATGGATGTCATTGTCTATACGAGATGCTCCTACGTGAAATCTGAAAGTTAACGGGAGGCATTATGATAGAATTTTTACGTAAGCTAATCCCTTGGGTTCTCGCTGGGATGCTATTCGGGTTAGGATGGAATCTAGGGTAAGACTCAATGGACGCTAAATGGAAACAGGAGGT‑‑ACAATGAGTACGTTAag                                                                                                                                                                                                                                                                                             <  1:344214/301‑1 (MQ=255)`  
                  `gCTGGTGCTCTTGAAGAGTACAAAGAGAAAGTCGGTGATAACGGTCTTACGGATGATGATATTTACACATTACAGTGATATACTCAAGGCCACTACAGATAGTGGTCTTTATGGATGTCATTGTCTATACGAGATGCTCCTACGTGAAATCTGAAAGTTAACGGGAGGCATTATGCTAGAATTTTTACGTAAGCTAATCCCTTGGGTTCTCGCTGGGATGCTATTCGGGTTAGGATGGCATCTAGGGTCAGACTCAATGGACGCTAAATGGAAACAGGAGGT‑‑ACAATGAGTACGTTAagag                                                                                                                                                                                                                                                                                           <  1:500001/301‑1 (MQ=255)`  
                  `gCTGGTGCTCTTGAAGAGTACAAAGAGAAAGTCGGTGATAACGGTCTTACGGATGATGATATTTACACATTACAGTGATATACTCAAGGCCACTACAGATAGTGGTCTTTATGGATGTCATTGTCTATACGAGATGCTCCGACGTGAAATCTGAAAGTTAACGGGAGGCATTATGCTAGAATTTTTACGTAAGCTAATCCCTTGGGTTCTCGCTGGGATGCTATTCGGGTTAGGATGGCATCTAGGGTCAGACTCAATGGACGCTAAATGGAAACAGGAGGT‑‑ACAATGAGTACGTTAagag                                                                                                                                                                                                                                                                                           <  1:115036/301‑1 (MQ=255)`  
                    `tGGTGTTTTTTAAGAGTAAAAATAGAAAGTCGGTGATAACGTTCTTACGTATGATGATATTTCACCATTAACGTGATTTACTCAATGCCACTACAGATATTGGTCTTTATGGATGTCATTGTCTTTACGAGATGCTCCTACGTGAAATCTGAAAGGTAACGGGAGTCATTATGCTAGAATTTTTACGTAAGCTAATCCCTTGGGTTCTCGCTGGGATGCTATTCGGGTTAGGATGGCATCTAGGGTCAGACTCAATGGACGCTAAATGGAAACAGGAGGTACACAATGAGTACGTTAagag                                                                                                                                                                                                                                                                                           <  1:151957/301‑1 (MQ=255)`  
                        `gCTCTTGAAGAGTACAAAGAGAAAGTCGGTGATAACGGTCTTACGGATGATGATATTTACACATTACAGTGATATACTCAAGGCCACTACAGATAGTGGTCTTTATGGATGTCATTGTCTATACGAGATGCTCCTACGTGAAATCTGAAAGTTAACGGGAGGCATTATGCTAGAATTTTTACGTAAGCTAATCCCTTGGGTTCTCGCTGGGATGCTATTCGGGTTAGGATGGCATCTAGGGTCAGTCTCAATGGACGCTAAATGGAAACAGGAGGTACACAATGAGTACgctgtctcttat                                                                                                                                                                                                                                                                                       >  1:160736/1‑290 (MQ=255)`  
                         `ctctTTAAGAGTACAAAGAGAAAGTCGGTGATAACGGTCTTACGGATGATGATATTTACACATTACAGTGATATACTCAAGGCCACTACAGATAGTGGTCTTTATGGATGTCATTGTCTATACGAGATGCTCCTACGTGAAATCTGAAAGTTAACGGGAGGCATTATGCTAGAATTTTTACGTAAGCTAATCCCTTGGGTTCTCGCTGGGATGCTATTCGGGTTAGGATGGCATCTAGGGTCAGACTCAATGGACGCTAAATGGAAACAGGAGGTACACAATgctgtctcttatacacatc                                                                                                                                                                                                                                                                                      >  1:146038/1‑283 (MQ=255)`  
                         `ctctTGAAGAGTACAAAGAGAAAGTCGGTGATAACGGTCTTACGGATGATGATATTTACACATTACAGTGATATACTCAAGGCCACTACAGATAGTGGTCTTTATGGATGTCATTGTCTATACGAGATGCTCCTACGTGAAATCTGAAAGTTAACGGGAGGCATTATGCTAGAATTTTTACGTAAGCTAATCCCTTGGGTTCTCGCTGGGATGCTATTCGGGTTAGGATGGCATCTAGGGTCAGACTCAATGGACGCTAAATGGAAACAGTAGGT‑‑ACAATGAGTACGTTAAGAGAGTTGAg                                                                                                                                                                                                                                                                                    >  1:31304/1‑301 (MQ=255)`  
                                `agagTACAAAGAGAAAGTCGGTGATAACGGTCTTACGGATGATGATATTTACACATTACAGTGATATACTCAAGGCCACTACAGATAGTGGTCTTTATGGATGTCATTGTCTATACGAGATGCTCCTACGTGAAATCTGAAAGTTAACGGGAGGCATTATGCTAGAATTTTTACGTAAGCTAATCCCTTGGGTTCTCGCTGGGATGCTATTCGGGTTAGGATGGCATCTAGGGTCAGACTCAATGGACGCTAAATGGAAACAGGAGGT‑‑ACAATGAGTACGTTAagagctgtctcttataca                                                                                                                                                                                                                                                                             >  1:22648/1‑287 (MQ=255)`  
                                   `gTACAAAGAGAAAGTCGGTGATAACGGTCTTACGGATGATGATATTTACACATTACAGTGATATACTCAAGGCCACTACAGATAGTGGTCTTTATGGATGTCATTGTCTATACGAGATGCTCCTACGTGAAATCTGAAAGTTAACGGGAGGCATTATGCTAGAATTTTTACGTAAGCTAATCCCTTGGGTTCTCGCTGGGATGCTATTCGGGTTAGGATGGCATCTAGGGTCAGACTCAATGGACGCTAAATGGCAACAGGAGGT‑‑ACAATGAGTACGTTAAGAGAGTTGAGGCTGCGAaga                                                                                                                                                                                                                                                                          >  1:293376/1‑301 (MQ=255)`  
                                          `gagaAAGTCGGTGATAACGGTCTTACGGATGATGATATTTACACATTACAGTGATATACTCAAGGCCACTACAGATAGTGGTCTTTATGGATGTCATTGTCTATACGAGATGCTCCTACGTGAAATCTGAAAGTTAACGGGAGGCATTATGCTAGAATTTTTACGTAAGCTAATCCCTTGGGTTCTCGCTGGGATGCTATTCGGGTTAGGATGGCATCTAGGGTCAGACTCAATGGACGCTAAATGGAAACAGGAGGT‑‑ACAATGAGTACGTTAAGAGAGTTGAGGCTGCGAAGAGCACTCa                                                                                                                                                                                                                                                                   >  1:19360/1‑301 (MQ=255)`  
                                                     `agATAACTGTATTACGGATGATGATATTGACACAGTACAGGGATATACTCAAGGCCAATACAGATAGTGGTCTTTATGGATGTCATTGTATATACGAGATGCTCCTACGTGAAATCTGAAAGGTAACGGGAGGCATTATGCTAGAATTTTTACGTAAGCTAATCCCTTGGGTTCTCGCTGGGATGCTATTCGGGTTAGGATGGCATCTAGGGTCAGACTCAATGGACGCTAAATGGAAACAGGAGGT‑‑ACAATGAGTACGTTAAGAGAGTTGAGGCTGCGAAGAGCACTCAAAGAGCAATCg                                                                                                                                                                                                                                                        <  1:137028/300‑1 (MQ=255)`  
                                                          `aCGGTCTTACGGATGATGATATTTACACATTACAGTGATATACTCAAGGCCACTACAGATAGTGGTCTTTATGGATGTCATTGTCTATACGAGATGCTCCTACGTGAAATCTGAAAGTTAACGGGAGGCATTATGCTAGAATTTTTACGTAAGCTAATCCCTTGGGTTCTCGCTGGGATGCTATTCGGGTTAGGATGGCATCTAGGGTCAGACTCAATGGACGCTAAATGGAAACAGGAGGT‑‑ACAATGAGTACGTTAAGAGAGTTGAGGCTGCGAAGAGCACTCAACGAGCAATCGATGCg                                                                                                                                                                                                                                                   >  1:402897/1‑301 (MQ=255)`  
                                                           `tggTCTTACGTATGATGAAATTTACAACTTACAGTGATATACTCAAGGCCACTACAGATAGTGGTCTTTATGGATGTAATTGTCTATACGAGATTCTCCTACGTGAAATCTGAAAGTTAACGGGAGGCATTATGCTAGAATTTTTACGTAAGCTAATCCCTTGGGTTCTCGCTGGGATGCTATTCGGGTTAGGATGGCATCTAGGGTCAGACTCAATGGACGCTAAATGGAAACAGGAGGT‑‑ACAATGAGTACGTTAAGAGAGTTGAGGCTGCGAAGAGCACTCAAAGAGCAATCGATGCgg                                                                                                                                                                                                                                                  <  1:66121/300‑1 (MQ=255)`  
                                                           `cGGTCTTACGGATGATGATATTTACACATTACAGTGATATACTCAAGGCCACTACAGATAGTGGTCTTTATGGATGTCATTGTCTATACGAGATGCTCCTACGTGAAATCTGAAAGTTAACGGGAGGCATTATGCTAGAATTTTTACGTAAGCTAAACACTTGGGTTCTCGATGGGATGCTATTCGGATTAGGATGGAATCTAGGGTCAGACTCAATGGACGCTAAATGGAAACAGGAGGTACACAATGAGTACGTTAATAGAGTTGAGGCTGCGAAGAGCACTCaactgtctcttataca                                                                                                                                                                                                                                                    >  1:8119/1‑287 (MQ=255)`  
                                                                `ttACGGATGATGATATTTACACATTACAGTGATATACTCAAGGCCACTACAGATAGTGGTCTTTATGGATGTCATTGTCTATACGAGATGCTCCTACGTGAAATCTGAAAGTTAACGGGAGGCATTATGCTAGAATTTTTACGTAAGCTAATCCCTTGGGTTCTCGCTGGGATGCTATTCGGGTTAGGATGGCATCTAGGGTCAGACTCAATGGACGCTAAATGGAAACAGGAGGT‑‑ACAATGTGTACGTTAAGAGAGTTGAGGCTGCGAAGAGCACTCAAAGAGCAATCGATGCggctgtc                                                                                                                                                                                                                                             >  1:278509/1‑296 (MQ=255)`  
                                                                  `tgtgtataagagacagacacATTACAGTGATATACTCAAGGCCACTACAGATAGTGGTCTTTATGGATGTCATTTTCTATACGATATGCTCCTACGTGAAATCTGAAAGTTAACGGGAGGCATTATGCTAGAATTTTTACGTAAGCTAATCCCTTGGGTTATCGCTGGGATGCTATTCGGGTTAGGATGGCATCTAGGGTCAGACTCAATGGACGCTAAATGGAAACAGGAGGT‑‑ACAATGAGTACGTTAAGAGAGTTGAGGCTGCGAAGAGCACTCAAAGAGCAATCGATGCGGTATCTGc                                                                                                                                                                                                                                           <  1:258871/285‑1 (MQ=255)`  
                                                                  `gtggatcgtagaggtgtataagatacaggATATCCTCAAGTCCACTCAAGATAGTGGTTTTTATTTATGTCATTGTCTATACGAGATGCTCCTACGTGAAATCTGAAAGTTAACTGGAGGCATTATGCTAGAATTTTTACGTAAGCTAATCCCTTGGGTTCTCGCTGGGATGCTATTCGGGTTAGGATGGCATCTAGGGTCAGACTCAATGGACGCTAAATGGAAACAGGAGGT‑‑ACAATGAGTACGTTAAGAGAGTTGAGGCTGCGAAGAGCACTCAAAGAGCAATCGATGCGGTATCTGc                                                                                                                                                                                                                                           <  1:110119/273‑1 (MQ=255)`  
                                                                    `ggATGTTGATATTTACACATTACAGTTATATACTCAAGGCCACTACAGATAGGTGTCTTTATGGATGTCATTTTCTATACGAGATGCTCCTACGTGAAATCTGAAAGTTAACGGGAGGCATTATGCTAGAATTTTTACGTAAGCTAATCCCTTGGGTTCTCGCTGGGATGCTATTCGGGTTAGGATGGCATCTAGGGTCAGACTCAATGGACGCTAAATGGAAACAGGAGGT‑‑ACAATGAGTACGTTAAGAGAGTTGAGGCTGCGAAGAGCACTCAAAGAGCAATAGATGCGGTATCTGCTa                                                                                                                                                                                                                                         <  1:174299/301‑1 (MQ=255)`  
                                                                     `gatgatgatATTTACACATTACAGTGATATACTCAAGGCCACTACAGATAGTGGTCTTTATGGATGTCATTGTCTATACGAGATGCTCCTACGTGAAATCTGAAAGTTAACGGGAGGCATTATGCTAGAATTTTTACGTAAGCTAATCCCTTGGGTTCTCGCTGGGATGCTATTCGGGTTAGGATGGCATCTAGGGTCAGACTCAATGGACGCTAAATGGAAACAGGAGGT‑‑ACAATGAGTACGTTAAGAGAGTTGAGGCTGCGAAGAGCACTCAAAGAGCAATCGATGCGGTATCTGCTaa                                                                                                                                                                                                                                        >  1:189442/1‑301 (MQ=255)`  
                                                                     `gatgatgatATTTACACATTACAGTGATATACTCAAGGCCACTACAGATAGTGGTCTTTATGGATGTCATTGTCTATACGAGATGCTCCTACGTGAAATCTGAAAGTTAACGGGAGGCATTATGCTAGAATTTTTACGTAAGCTAATCCCTTGGGTTCTCGCTGGGATGCTATTCGGGTTAGGATGGCATCTAGGGTCAGACTCAATGGACGCTAAATGGAAACAGGAGGT‑‑ACAATGAGTACGTTAAGAGAGTTGAGGCTGCGAAGAGCACTCAAAGAGCAATCGATGCGGTATCTGCTaa                                                                                                                                                                                                                                        >  1:422762/1‑301 (MQ=255)`  
                                                                            `atatTTACACATTACAGTGATATACTCAAGGCCACTACAGATAGTGGTCTTTATGGATGTCATTGTCTATACGAGATGCTCCTACGTGAAATCTGAAAGTTAACGGGAGGCATTATGCTAGAATTTTTACGTAAGCTAATCCCTCGGGATCTAGCTGGGATGCTATTCGGGTTAGGATGGCATCTAGGGTCAGACTCAATGGACGCTAAATGGAAACAGGAGGTACACAATGAGTACGTTACGAGAGTTGATGCTGCGAAGAGCACTCAAAGAGCAATCGATGCGGTATCTGCTAAGTATc                                                                                                                                                                                                                                   >  1:478104/1‑301 (MQ=255)`  
                                                                             `tatTTACACATTACAGTGATATACTCAATGCCACTACAGATAGTGGTCTTTATGGATGTCATTGTCTATACGAGATGCTCCTTCGTGAAATCTGAAAGTTAACGGGAGGCATTATGCTAGAATTTTTCCTTAATCTAATCCCTTGGGTTCTCTCTGGGATGCTATTTGGGTTAGGATTGCATCTAGGGTCAGACTCAAGGGACGCTAAATGGAAACAGGAGGT‑‑ACAATGAGTACGTTCAGAGAGTTGAGGCTGCGAAGAGCACTCAAAGCGCAATCGATGCGGTATCTGCTAAGTATCaaa                                                                                                                                                                                                                                >  1:124212/1‑300 (MQ=255)`  
                                                                             `tatTTACACATTACAGTGATATACTCAAGGTCACTACAGATAGTGGTCTTTATGGATGTCATTGTCTATACGAGATGCTCCTACGTGAAATCTGAAAGTTAACGGGAGGCATTATGCTAGAATTTTTACGTAAGCTAATCCCTTGGGTTCTCGCTGGGATGCTATTCGGGTTAGGATGGCATCTAGGGTCAGACTCAATGGACGCTAAATGGAAACAGGAGGT‑‑ACAATGAGTACGTTAAGAGAGTTGAGGCTGCGAAGAGCACTCAAAGAGCAATCGATGCGGTATCTGCTAAGTATCaaa                                                                                                                                                                                                                                >  1:408473/1‑300 (MQ=255)`  
                                                                              `atTTATACATTACAGTGATATACGCAAGGGCACTACAGATAGTTGTTTTTATGGATTTCATGGTATATACGATATGCTCCTACATTAAATATGAAAGTTAACGGGAGGCATTATGCTAGAATTTTTACGTAAGCTAATCCCTTGGGTTCTCGCTGGGATGCTATTCGGGTTAGCATGACATCTAGGGTCAGACTCAATGGACGCTAAATGGAAACAGGAGGT‑‑ACAATGAGTACGTTAAGAGAGTTGAGGCTGCGAAGAGCACTCAAAGAGCAATCGATGCGGTATCTGCTAAGTATCaaga                                                                                                                                                                                                                               <  1:62434/301‑1 (MQ=255)`  
                                                                              `atTTACACATTACAGTGATATACTCAAGGCCACTACAGATAGTGGTCTTTATGGATGTCATTGTCTATACGAGATGCTCCTACGTGAAATCTGAAAGTTAACGGGAGGCATTATGCTAGAATTTTTACGTAAGCTAATCCCTTGGGTTCTCGCTGGGATGCTATTCGGGTTAGGATGGCATCTAGGGTCAGACTCAATGGACGCTAAATGGAAACAGGAGGT‑‑ACAATGAGTACGTTAAGAGAGTTGAGGCTGCGAAGAGCACTCAAAGAGCAATCGATGCGGTATCTGCTAAGTATCaaga                                                                                                                                                                                                                               <  1:398316/301‑1 (MQ=255)`  
                                                                              `atTTACAAATTACAGTGATATACTCAAGGCAACTACAGATAGTGGTCTTTATGGATGGCATTGTCTATACGAGATGCTCCTACGTGAAATGTGAAAGTTAACGGGAGGCATTATGCTAGAATTTTTACGTAAGCTAATCCCTTGGGTTCTCGCTGGAATGCTATTCGGGTTAGGATGGCATCTAGGGTCAGACTCAATGGACGCTAAATGGAAACAGGAGGT‑‑ACAATGAGTACGTTAAGAGAGTTGAGGCTGCGAAGAGCACTCAAAGAGCAATCGATGCGGTATCTGCTAAGTATCaaga                                                                                                                                                                                                                               <  1:469050/301‑1 (MQ=255)`  
                                                                              `atTTAAACATTACATTGATATACTCAAGGCCACTACAGATAGTGGTCTTTATGGATGTCATTGTCTATACGAGATGCTCCTACGTGAAATCTGAAAGTTAACGGGAGGCATTATGCTAGAATTTTTACGTAAGCTAATCCCTTGGGTTCTCGCTGGGATGCTATTCGGGTTAGGATGGCATCTAGGGTCAGACTCAATGGACGCTAAATGGAAACAGGAGGT‑‑ACAATGAGTACGTTAAGAGAGTTGAGGCTGCGAAGAGCACTCAAAGAGCAATCGATGCGGTATCTGCTAAGTATCaaga                                                                                                                                                                                                                               <  1:94940/301‑1 (MQ=255)`  
                                                                                `gacagcaTTATAGTGATATACTCAAGGCCACTACAGATAGTGGTCTTTATGGATGTCATTGTCTATACGAGATGCTCCTAAGTGAAATCTGAAAGTTAACGGGAGGCATTATGCTAGAATTTTTACGTAAGCTAATCCCGTGGGGTCTCGCTGGGATGCTATTCGGGTTAGGATGGCATCTAGGGTCAGACTCAATGGACGCTAAATGGAAACAGGAGGT‑‑ACAATGAGTACGTTAAGAGAGTTGAGGCTGCGAAGAGCACTCAAAGAGCAATCGATGCGGTATCTGCTAAGTATCaagaag                                                                                                                                                                                                                             <  1:426981/296‑1 (MQ=255)`  
                                                                                  `acacATTACAGTGATATACTCAAGGCCACTACAGATAGTGGTCTTTATGGATGTCATTGTCTATACGAGATGCTCCTACGTGAAATCTGAAAGTTAACGGGAGGCATTATGCTAGAATTTTTACGTAAGCTAATCCCTTGGGTTCTCGCTGGGATGCTATTCGGGTTAGGATGGCATCTAGGGTCAGACTCAATGGACGCTAAATGGAAACAGGAGGT‑‑ACAATGAGTACGTTAAGAGAGTTGAGGCTGCGAAGAGCACTCAAAGAGCAATCGATGCGGTATCTGCTAAGTATCaagactgt                                                                                                                                                                                                                           >  1:459008/1‑297 (MQ=255)`  
                                                                                    `acaTTACAGTGATATACTCAAGGCCACTACAGATAGTGGTCTTTATGGATGTCATTGTCTATACGAGATGCTCCTACGTGAAATCTGAAAGTTAACGGGAGGCATTATGCTAGAATTTTTACGTAAGCTAATCCCTTGGGTTCTCGCTGGGATGCTATTCGGGTTAGGATGGCATCTAGGGTCAGACTCAATGGACGCTAAATGGAAACAGGAGGT‑‑ACAATGAGTACGTTAAGAGAGTTGAGGCTGCGAAGAGCACTCAAAGAGCAATCGATGCGGTATCTGCTAAGTATCAAGAAGACCt                                                                                                                                                                                                                         >  1:274385/1‑301 (MQ=255)`  
                                                                                     `caTTACAGTGATATACTCAAGGCCACTACAGATAGTGGTCTTTATGGATGTCATTGTCTATACGAGATGCTCCAACGTTAAATCTGAAAGTTAACGGGAGGCATTATGCTAGAATTTTTACGTACGCTAATCCCTTGGGTTATAGCTGGGATGCTATTCGGGTTAGGATGGCACTTAGGGTCAGACTCAATGGATGCTAAATGGAAACAGGAGGT‑‑ACAATGAGTACGTTAAGAGAGTTGAGGCTGCGAAGAGCACTAAAAGAGCAATAGATGCGGTATCTGATAAGTATCaaacagaacat                                                                                                                                                                                                                        >  1:504912/1‑292 (MQ=255)`  
                                                                                       `ttACAGTGATATACTCAAGGCCACTACAGATAGTGGTCTTTATGGATGTCATTGTCTATACGAGATGCTCCTACGTGAAATCTGAAAGTTAACGGGAGGCATTATGCTAGAATTTTTACGTAAGCTAATCCCTTGGGTTCTCGCTGGGATGCTATTCGGGTTAGGATGGCATCTAGGGTCAGACTCAATGGACGCTAAATGGAAACAGGAGGT‑‑ACAATGAGTACGTTAAGAGAGTTGAGGCTGCGAAGAGCACTCAAAGAGCAATCGATGCGGTATCTGCTAAGTATCAAGAAGACCTTGc                                                                                                                                                                                                                      >  1:372327/1‑301 (MQ=255)`  
                                                                                            `gTGATATACTCAAGGCCACTACAGATAGTGGTCTTTATGGATGTCATTGTCTATACGAGATGCTCCTACGTGAAATCTGAAAGTTAACGGGAGGCATTATGCTAGAATTTTTACGTAAGCTAATCCCTTGGGTTCTCGCTGGGATGCTATTCGGGTTAGGTTGGCATCTAGTGTCAGACTCAATGGACGCTAAATGGAAACAGGAGGT‑‑ACAATGAGTACGTTAAGAGAGTTTAGGCTGCGAAGAGCACTAAAAGAGCAATCGATGCGGTATCTGCTAAGTATCAAGAAGACCTTGCCgcgc                                                                                                                                                                                                                 >  1:285764/1‑301 (MQ=255)`  
                                                                                              `gATATACTCAAGGCCACTACAGATAGTGGTCTTTATGGATGTCATTGTCTATACGAGATGCTCCTACGTGAAATCTGAAAGTTAACGGGAGGCATTATGCTATAATTTTTACGTAAGCTAATCCCTTGGGTTCTCGCTGGGATGCTATTCGGGTTAGGATGGCATCTAGGGTCAGACTCAATGGACGCTAAATGGAAACAGGAGGT‑‑ACAATGAGTACGTTAAGAGAGTTGAGGCTGCGAAGAGCACTCAAAGAGCAATCGATGCGGTATCTGCtctgtctcttatacacatctccgagccc                                                                                                                                                                                                               >  1:184073/1‑274 (MQ=255)`  
                                                                                                   `aCTCAAGGCCACTACAGATAGTGGTCTTTATGGATGTCATTGTCTATACGAGATGCTCCTACGTGAAATCTGAAAGTTAACGGTAGGCATTATGCTAGAATTTTTACGTAAGCTAATCCCTTGGGTTCTCGCTGGGATGCTATTCGGGTTAGGATGGCATCTAGGGTCAACCTCAATGGACGCGAAATGGAAACAGGAGGTACACAATGAGTACGTTAAGAGAGTTGAGGCTGCGAAGAGCACTCAACGAGCAATCGATGCGGAATCTGCTAAGTATCAAGAAGACCTTGCCGCGCTGGaa                                                                                                                                                                                                            >  1:105680/1‑301 (MQ=255)`  
                                                                                                       `ttataagagaCAGATAGTGGTCTTTATGGATGTCATTGTCTATACGAGATGCGCATACGTGAAATCTGAAAGTTAACGGGAGGCATTATGCTAGAATTTTTACGTAAGCTAATCCCTTGGGTTCTCGCTGGGATGCTATTCGGGTTAGGATGGCATCTAGGGTCAGACTCAATGGACGCTAAATGGAAACAGGATGT‑‑ACAATGAGTACGTTAAGAGAGTTGAGGCTGCGAAGAGCACTCAAAGAGCAATCGATGCGGTATCTGCTAAGTATCAAGAAGACCTTGCCGCGCTGGAAGGGAGc                                                                                                                                                                                                      <  1:75934/292‑1 (MQ=255)`  
                                                                                                       `aaGGCCACTACAGATAGTGGTCTTTATGGATGTCATTGTCTATACGAGATGCTCCTACGTGAAATCTGAAAGTTAACGGGAGGCATTATGCTAGAATTTTTACGTAAGCTAATCCCTTGGGTTCTCGCTGGGATGCTATTCGGGTTAGGATGGCATCTAGGGTCAGACTCAATGGACGCTAAATTGAAACAGGAGGT‑‑ACAATGAGTACGTTAAGAGAGTTGAGGCTGCGAAGAGCACTCAAAGAGCAATCGATGCGGTATCTGCTAAGTATCAAGAAGACCTTGCCGCGCTGGAAGGGAGc                                                                                                                                                                                                      <  1:483569/301‑1 (MQ=255)`  
                                                                                                         `ggCCACTACAGATAGTGGTCTTTATGGATGTCATTGTCTATACGAGATGCTCCTACGTGAAATCTGAAAGTTAACGGGAGGCATTATGCTAGAATTTTTACGTAAGCTAATCCCTTGGGTTCTCGCTGGGATGCTATTCGGGTTAGGATGGCATCTAGGGTAAGACTCAATGGACGCTAAATGGAAACAGGAGGT‑‑ACAATGAGTACGTTAAGAGAGTTGAGGCTGCGAAGAGCACTCAAAGAGCAATCGATGCGGTATCTGCTAAGTATCaagactgtatcttatacaaatctccgagccc                                                                                                                                                                                                    >  1:336823/1‑274 (MQ=255)`  
                                                                                                           `aaaaggcAGATGTTGGTCTTTATGGAGGTCATTTGCTATACGAGATGCTCCTACGTGAAATCTGAAAGGTAACGGGAGGCATTATGCTAGAATTTTTACGTAAGCTAATCCCTTGGGTTCTCGCTGGGATGCTATTCGGGTTAGGATGGCATCTAGGGTCAGACTCAATGGACGCTAAATGGAAACAGGAGGT‑‑ACAATGAGTACGTTAAGAGAGTTGAGGCTGCGAAGAGCACTCAAAGAGCAATCGATGCGGTATCTGCTAAGTATCAAGAAGACCTTGCCGCGCTGGAAGGGAGCACTg                                                                                                                                                                                                  <  1:303169/295‑1 (MQ=255)`  
                                                                                                            `cACGACAGATAGTGGTCTTTAGGGATGTCATTGTCTATACTGGATGCTCCTTCGTGAAATCTGAAAGTTAACGGGATGCATTATGCTAGAATTTTTACGTAAGCTAATCCCTTGGGTTCTCGCTGGGATGCTATTCGGGTTAGGATGGCATCTAGGGTCAGACTCAATGGACGCTAAATGGAAACAGGAGGT‑‑ACAATGAGTACGTTAAGAGAGTTGAGGCTGCGAAGAGCACTCAAAGAGCAATCGATGCGGTATCTGCTAAGTATCAAGAAGACCTTGCCGCGCTGGAAGGGAGCACTGa                                                                                                                                                                                                 <  1:41343/301‑1 (MQ=255)`  
                                                                                                              `cTACAGATAGTGGTCTTTATGGATGTCTTTTTCTATACGAGATGCTCCTACGTTAAATCTAAAATTTTACGGGAGGCATTATGCTAGAATTTTTCCGTAAGCTAATCCCTTGTGTTCTCGCTGGTATGCTATTCGGGTTAGGATGGCCTCTAGGGTCAGACTCAATGGACGCTAAATGGACACAGGAGTTA‑TC‑ATGAGTACGTTAAGATAGTTGAGGCTGCGAAGAGCACTCAAAGAGCAATCGATGCGGTATCTGCTAAGTATCAAGAAGACCTTGCCGCGCTGGAAGGGAGCACTGATa                                                                                                                                                                                               <  1:43619/301‑1 (MQ=255)`  
                                                                                                              `cTACAGATAGTGGTCTTTATGGATGTCATTGTCTATACGAGATGCTCCTACGTGAAATCTGAAAGTTAACGGGAGGCATTATGCTAGAATTTTTACGTAAGCTAATCCCTTGGGTTCTCGCTGGGATGCTATTCGGGTTAGGATGGCATCTAGGGTCAGACTCAATGGACGATAAATGGAAACAGGAGGTACACAATGAGTACGTTAAGAGAGTTGAGGCTGCGAAGAGCACTCAAAGAGCAATCGATGCGGTATCTGCTAAGTATCAAGAAGACCTTGCCGCGCTGGAAGGGCGCACTGa                                                                                                                                                                                                 >  1:141178/1‑301 (MQ=255)`  
                                                                                                                 `cAGATAGTTGTCTTTATGGATGTCATTGTCTATACGAGATGCTCCTACGTGAAATCTGAAAGTTAACGGGAGGCATTATGCTAGAATTTTTACGTAAGCTAATCCCTTGGGTTCTCGCTGGGATGCTATTCGGGTTAGGATGGCATCTAGGGTCAGACTCAATGGACGCTAAATGGAAACAGGAGGT‑‑ACAATGAGTACGTTAAGAGAGTTGAGGCTGCGAAGAGCACTCAAAGAGCAATCGATGCGGTATCTGCTAAGTATCAAGAAGACCTTGCCGCGCTGGAAGGGAGCACTGATAGGa                                                                                                                                                                                            >  1:155539/1‑301 (MQ=255)`  
                                                                                                                   `gATAGTGGTCTTTATGGATGTCATTGTCTATACGAGATGCTCCTACGTGAAATCTGAAAGTTAACGGGAGGCATTATGCTAGAATTTTTACGTAAGCTAATCCCTTGGGTTCTCGCTGGGATGCTATTCGGGTTAGGATGGCATCTAGGGTCAGACTCAATGGACGCTAAATGGAAACAGGAGGT‑‑ACAATGAGTACGTTAAGAGAGTTGAGGCTGCGAAGAGCACTCAAAGAGCAATCGATGCGGTATCTGCTAAGTATCAAGAAGACCTTGCGGCGCTGGAAGGGAGCACTgctgtctct                                                                                                                                                                                          >  1:494070/1‑293 (MQ=255)`  
                                                                                                                   `aaTAGTGGTCTTTATGGATGTCATTGTCGATACGAGATGCTCCTACGTGAAATCTGAAAGTTAACGGGAGGCATTATGCTAGAATTTTTACGTAAGCTAATCCCTTGGGTTCTCGCTGGGATGCTATTCGGGTTAGGATGGCATCTAGGGTCAGACTCAATGGACGCTAAATGGAAACAGGAGGTACACAATGAGTACGTTAAGAGAGTTGAGGCTGCGAAGAGCACTCAAAGAGCAATCGATGCGGTATCTGCTAAGTATCAAGAAGACCTTGCCGCGCTGGAAGGGAGCACTGATAGGa                                                                                                                                                                                            <  1:309240/300‑1 (MQ=255)`  
                                                                                                                       `gTGGTCTTTATGGATGTCATTGTCTATACGAGATGCTCCTACGTGAAATCTGAAAGTTAACGGGAGGCATTATGCTAGAATTTTTACGTAAGCTAATCCCTTGGGTTCTCGCTGGGATGCTATTCGGGTTAGGATGGCATCTAGGGTCAGACTCAATGGACGCTAAATGGAAACAGGAGGTACACAATGAGTACGTTAAGAGAGTTGAGGCTGCGAAGAGCACTCAAAGAGCAATCGATGCGGTATCTGCTAAGTATCAAGAAGACCTTGCCGCGCTGGAAGGGAGCACTGATAGGattat                                                                                                                                                                                        >  1:376371/1‑301 (MQ=255)`  
                                                                                                                       `gTGGTCTTTATGGATGTCATTGTCTATACGAGATGCTCCTACGTGAAATCTGAAAGTTAACGGGAGGCATTATGCTAGAATTTTTACGTAAGCTAATCCCTTGGGTTCTCGCTGGGATGCTATTCGGGTTAGGATGGCATCTAGGGTCAGACTCAATGGACGCTAAATGGAAACAGGAGGT‑‑ACAATGAGTACGTTAAGAGAGTTGAGGCTGCGAAGAGCACTCAAAGAGCAATCGATGCGGTATCTGCTAAGTATCAAGAAGACCTTGCCGCGCTGGAAGGGAGCACTGATAGGATTAttt                                                                                                                                                                                      >  1:119576/1‑301 (MQ=255)`  
                                                                                                                         `ggTCTTTATGGATGTCATTGTCTATACGAGATGCTCCTACGTGAAATCTGAAAGTTAACGGGAGGCATTATGCTAGAATTTTTACGTAAGCTAATCCCTTGGGTTCTCTCTGGGATGCTATTCGGGTTAGGATGGCATCTAGGGTCAGACTCAATGGACGCTAAATGGAAACAGGAGGT‑‑ACAATGAGTACGTTAAGAGAGTTGAGGCTGGGAAGAGCACTCAAAGAGCAATCGATGAGGTAGCTGCTAAGTATCAAGAAGACCTTGCCGCGCTGGAAGGGAGCGCTGATAGGATTATTTCt                                                                                                                                                                                    >  1:332589/1‑301 (MQ=255)`  
                                                                                                                           `tCTTTATGGATGTCATTGTCTATACGAGATGCTCCTACGTGAAATCTGAAAGTTAACGGGAGGCATTATGCTAGAATTTTTACGTAAGCTAATCCCTTGGGTTCTCGCTGGGATGCTATTCGGGTTAGGATGGCATCTAGGGTCAGACTCAATGGACGCTAAATGGAAACAGGAGGTACACAATGAGTACGTTAAGAGAGTTGAGGCTGCGAAGAGCACTCAAAGAGCAATCGATGCGGTATCTGCTAAGTATCAAGAAGACCTTGCCGCGCTGGAAGGGAGCACTGATAGGatctgcctc                                                                                                                                                                                    >  1:231168/1‑294 (MQ=255)`  
                                                                                                                                            `gTCTATACGAGATGCTCCTACGTGAAATCTGAAAGTTAACGGGAGGCATTATGCTAGAATTTTTACGTAAGCTAATCCCTTGGGTTCTCGCTGGGATGCTATTCGGGTTAGGATGGCATCTAGGGTCAGACTCAATGGACGCTAAATGGAAACAGGAGGT‑‑ACAATGAGTACGTTAAGAGAGTTGAGGCTGCGAAGAGCACTCAAAGAGCAATCGATGCGGTATCTGCTAAGTATCAAGAAGACCTTGCCGCGCTGGAAGGTAGCACTGATAGGATTATTTCTGATTTGCGTAGCGActgtc                                                                                                                                                                 >  1:418474/1‑297 (MQ=255)`  
                                                                                                                                            `gTCTATACGAGATGCTCCTACGTGAAATCTGAAAGTTAACGGGAGGCATTATGCTAGAATTTTTACGTAAGCTAATCCCTTGGGTTCTCGCTGGGATGCTATTCGGGTTAGGATGGCATCTAGGGTCAGACTCAATGGACGCTAAATGGAAACAGGAGGT‑‑ACAATGAGTACGTTAAGAGAGTTGAGGCTGCGAAGAGCACTCAAAGAGCAATCGATGCGGTATCTGCTAAGTATCAAGAAGACCTTGCCGCGCTGGAAGGGAGCACTGATAGGATTATTTCTGAttctgtctcttatacac                                                                                                                                                                 >  1:340934/1‑286 (MQ=255)`  
                                                                                                                                                    `gagaTGCTCCTACGTGAAATCTGAAAGTTAACGGGAGGCATTATGCTAGAATTTTTACGTAAGCTAATCCCTTGGGTTCTCGCTGGGATGCTATTCGGGTTAGGATGGCATCTAGGGTCAGACTCAATGGACGCTAAATGGAAACAGGAGGT‑‑ACAATGAGTACGTTAAGAGAGTTGAGGCTGCGAAGAGCACTCAAAGAGCAATCGATGCGGTATCTGCTAAGTATCAAGAAGACCTTGCCGCGCTGGAAGGGAGCACTGATGGGATTATTTCTGATTTGCGTAGCGACAATAAGCGGTTg                                                                                                                                                         >  1:56988/1‑301 (MQ=255)`  
                                                                                                                                                            `ccTACGTGAAATCTGAAAGTTAACGGGAGGCATTATGCTAGAATTTTTACGTAAGCTAATCCCTTGGGTTCTCGCTGGGATGCTATTCGGGTTAGGATGGCATCTAGGGTCAGACTCAATGGACGCTAAATGGAAACAGGAGGTACACAATGAGTACGTTAAGAGAGTTGAGGCTGCGAAGAGCACTCAAAGAGCAATCGATGCGGTATCTGCTAAGTATCAAGAAGACCTTGCCGCGCTGGAAGGGAGCACTGATATGATTATTTCTGATTTGAGTAGCGACAATAAGCGGTTGCGCGtt                                                                                                                                                   >  1:327625/1‑300 (MQ=255)`  
                                                                                                                                                                `cGTGAAATCTGAAAGTTAACGGGAGGCATTATGCTAGAATTTTTACGTAAGCTAATCCCTTGGGTTCTCGCTGGGATGCTATTCGGGTTAGGATGGCATCTAGGGTCAGACTCAATGGACGCTAAATGGAAACAGGAGGT‑‑ACAATGAGTACGTTAAGAGAGTTGAGGCTGCGAAGAGCACTCAAAGAGCAATCGATGCGGTATCTGCTAAGTATCAAGAAGACCTTGCCGCGCTGGAAGGGAGCACTGATAGGATTATTTCTGATTTGCGTAGCGACAATAAGCGGTTGCGCGTCAGAGTc                                                                                                                                             >  1:268086/1‑301 (MQ=255)`  
                                                                                                                                                                     `tagaggcaggTTAACGGGAGGCATTATGCTAGAATTTTTACGTAAGCTAATCCCTTGGGGTCTCGCTGGGATGCTATTCGGGTTAGGAAGGCATCTTGGGTAAGGCTCAATGGACGCTAAATGGAAACAGGAGGT‑‑ACAATGAGTACGTTAAGAGAGTTGAGGCTGCGAAGAGCACTCAAAGAGCAATCGATGCGGTATCTGCTAAGTATCAAGAAGACCTTGCCGCGCTGGAAGGGAGCACTGATAGGATTAGTTCTGATTTGCGTAGCGACAATAAGCGGTTGCGCGTCAGAGTCAAAAc                                                                                                                                        <  1:2408/292‑1 (MQ=255)`  
                                                                                                                                                                      `aTCTGAAAGTTAACGGGAGTCATTATGTATGAATTTTTACGGAAGCTAATTCCTTGGGTTCTAGCTGGGATGCTATTCGGTTTAGGATTGCATCTAGGGTCAGAATCAATGGACGCTAAATGGAAACAGGAGGT‑‑ACAATGAGTACGTTAAGAGAGTTGAGGCTGCGAAGAGCACTAAAAGAGCAATCGATGCGGTATCTGCTAAGTATCAAGAAGACCTTGCCGCGCTGGAAGGGAGCACTGATAGGATTATTTCTGATTTGCGTAGCGACAATAAGCGGTTGCGCGTCAGAGTCAAAACt                                                                                                                                       <  1:100015/301‑1 (MQ=255)`  
                                                                                                                                                                             `aGTTAACGGGAGGCATTATGCTAGAATTTTTACGTAAGCTAATCACTTGGGTTCTCACTGGGATGCTATTCGGGTTAGGATGGCATCTAGGGTCAGACTCAATGGACGCTAAATGGAAACAGGAGGT‑‑ACAATGAGTACGTTAAGAGAGTTGAGGCTGCGAAGAGCACTCAAAGAGCAATCGATGCGGTATCTGCTAAGTATCAAGAAGACCTTGCCGCGCTGGAAGGGAGCACTGATAGGA‑TATTTCTGATTTGCGTAGCGACAATAAGCGGTTGCGCGTCAGAGTCAAAACTACCGGAAc                                                                                                                               <  1:221313/301‑1 (MQ=255)`  
                                                                                                                                                                                         `agagacagCTAGAAGTTTTACGTAAGCTAATCCCTGGGGTTCTCGCTGTGATGCTATTCGGGTTAGGAGGGCATCTAGGGTCAGACTCAATGGACGCTAAATGGAAACAGGAGGT‑‑ACAATGAGTACGTTAAGAGAGTTGAGGCTGCGAATAGCACTCAAAGAGCAATCGATGCGGTATCTGCTAAGTATCAAGAAGACCTTGCCGCGCTGGAAGGGAGCACTGATAGGATTATTTCTGATTTGCGTAGCGACAATAAGCGGTTGCGCGTCAGAGTCAAAACTACCGGAACCTCCGATGGTc                                                                                                                    <  1:38592/294‑1 (MQ=255)`  
                                                                                                                                                                                             `tATGCTATAATTTTTACGTAAGCTAATCCCTTGGGTTCTCGGTGGGATGCTATTCGGGTTAGGATGGCAACTAGGGGTAGACTCAATTGACGCTAAATGGAAACAGGAGGT‑‑ACAATGAGTACGTTAAGAGAGTTGAGGCTGCGAAGAGCAATCAAAGAGCAATCGATGCGGTATCTGCTAAGTATCAAGAAGACCTTGCCGCGCTGGAAGGGAGCACTGATAGGATTATTTCTGATTTGCGTAGCGACAATAAGCGGTTGCGCGTCAGAGTCAAAACTACCGGAACCTCCGATGGTCAgtg                                                                                                                <  1:454464/301‑1 (MQ=255)`  
                                                                                                                                                                                              `aTGCTAGAATTTTTACGTAAGCTAATCCCTTGGGTTCTCGCTGGGATGCTATTCGGGTTAGGATGGCATCTAGGGTCTGACTCAATGGTCGCTAAATGGAAACAGGAGGT‑‑ACAATGATTACGTTAAGAGATTTTAGGCTGCTAAGCTCACTCAAATATCAATCGATGCGGTATCTGCTAAGTATCAAGAAGACCTTGCCTCGCTGCAAGGGAGCACTGATAGGTTTTTTTCTGATTTGCTTAGCGACTATTATCGGTTGCTCGTCATATTCATAACTACCGGAACTTCTGATGGTCatttt                                                                                                               >  1:262620/1‑297 (MQ=255)`  
                                                                                                                                                                                              `aTGCTAGAATTTTTACGTAAGCTAAGCCCTTGGGTTCTCGCTGGGATGCTATTCGGGTTAGGATGGCATCTAGGGTCAGACTCAATGGACGCTAAATGGAAACAGGAGGT‑‑ACAATGAGTACGTTAAGAGAGTTGAGGCTGCGAAGAGCACTCAAAGAGCAATCGATGCGGTATCTGCTAAGTATCAAGAAGACCTTGCCGCGCTGGAAGGGAGCACTGATAGGATTATTTCTGATTTGCGTAGCGACAATAAGCGGTTGCGCGTCAGAGTCAAAACTACCGGAACCTCCGATGGTCAgtgt                                                                                                               <  1:7083/301‑1 (MQ=255)`  
                                                                                                                                                                                                   `aGAATTTTTACGTAAGCTAATCCCTTGGGTTCTCGCTGGGATGCTATTCGGGTTAGGATGGCATCTAGGGTCAGACTCAATGGACGCTAAATGGAAACAGGAGGT‑‑ACAATGAGTACGTTAAGAGAGTTGAGGCTGCGAAGAGCACTCAAAGAGCAATCGATGCGGTATCTGCTAAGTATCAAGAAGACCTTGCCGCGCTGGAAGGGAGCACTGATAGGATTATTTCTGATTTGCGTAGCGACAATAAGCGGTTGCGCGTCAGAGTCAAAACTACCGGAACCTCCGATGGTCAGTGTGGAtt                                                                                                          <  1:363820/301‑1 (MQ=255)`  
                                                                                                                                                                                                            `gacagaGCTAATCCCTTGGGTTCTCGCTGTGATGCTATTCGGGTTAGGATGGCATCTAGTGTCAGACTCAATGGACGCTAAATGGAAACAGGAGGT‑‑ACAATGAGTACGTTAAGAGAGTTGAGGCTGCGAAGAGCACTCAAAGAGCAATCGATGCGGTATCTGCTAAGTATCAAGAAGACCTTGCCGCGCTGGAAGGGAGCACTGATAGGATTATTTCTGATTTGCGTAGCGACAATAAGCGGTTGCGCGTCAGAGTCAAAACTACCGGAACCTCCGATGGTCAGTGTGGATTCGAGCCTGa                                                                                                 <  1:367560/296‑1 (MQ=255)`  
                                                                                                                                                                                                            `aCGTACGCTAATCCCTTGGGTTCTCGCTGGGATGCTATTCGGGTTAGGATGGCATCTAGGGTCAGACTCAATGGACGCTAAATGGAAACAGGAGGT‑‑ACAATGAGTACGTTAAGAGAGTTGAGGCTGCGAAGAGCACTCAAAGAGCGATCGATGCGGTATCTGCTAAGTATCAAGACGACCTTGCCGCGCTGGAAGGGAGCACTGATAGGATTATTTCTGATTTGCGTAGCGACAATAAGCGGTTGCGCGTCAGAGTCAAAACTACCGGAACCTCCGATGTTCAGTTTGGATTCGAGCCTgc                                                                                                 >  1:509207/1‑300 (MQ=255)`  
                                                                                                                                                                                                               `agagacagtCCCTTGGGTTCTCGCTGGGATGCTATTCGGGTTAGTATGGCATCGAGGGTCATACTCAATGGACGCTAAATGGAAACAGGAGGT‑‑ACAATGAGTACGTTAAGAGAGTTGAGGCTGCGAAGAGCACTCAAAGAGCAATCGATGCGGTATCTGCTAAGTATCAAGAAGACCTTGCCGCGCTGGAAGGGAGCACTGATAGGATTATTTCTGATTTGCGTAGCGACAATAAGCGGTTGCGCGTCAGAGTCAAAACTACCGGAACCTCCGATGGTCAGTGTGGATTCGAGCCTGATgg                                                                                              <  1:16471/293‑1 (MQ=255)`  
                                                                                                                                                                                                                 `aGCTAATCCCTTGTGGTCTCGCTGGGATGCTATTCGGGTTAGGATGGCATCTAGGGTCAGACTCAATGGACGCTAACTGGAAACAGGAGGT‑‑ACAATGAGTACGTTAAGAGAGTTGAGGCTGCGAAGAGCACTCAAAGAGCAATCGATGCGGTATCTGCGAAGTATCAAGAAGACCTTGCCGCGCTGGAAGGGATCACTGATAGGATTATTTCTGATTTGCGTAGCGACAATAAGCGGTTGCGCGTCAGAGTCCAAACTACAGACACCTCCGATGGTCAGTGTGGAttatgtctcttataca                                                                                            >  1:53189/1‑287 (MQ=255)`  
                                                                                                                                                                                                                 `aGCTAATCCCTTGGGATCTCGCTGGGATGCTATTCGGGTTAGGATGGCATCTAGGGTCAGACTCAAGGGACGCTAAATGGAAACAGGAGGT‑‑ACAATGAGTACGATAAGAGAGTTGAGGCTGTGAAGAGCACTCAAAGAGCAATCGATGCGGTATCTGCTCAGTATCAAGCAGACCTTGCCGAGCTGGAAGGGAGCACTGATAGGATTATTTCTGATTTGCGTAGAGACACTAAGAGGTGGCGCGTCAGAGTCAAAACTACCGGAACCTCCGATGGTCAGTGTGGATTCGAGCATGATGGTc                                                                                            >  1:318437/1‑301 (MQ=255)`  
                                                                                                                                                                                                                                  `ctcGCTGGGATGCTATTCGGGTTAGGATGGCATCTAGGGTCAGACTCAATGGACGCTAAATGGAAACAGGAGGT‑‑ACAATGAGTACGTTAAGAGAGTTGAGGCTGCGAATAGCACTCAAAGAGCAATCGATGCGGTATCTGCTAAGTATCAAGAAGACCTTGCCGCGCTGGAAGGGAGCACTGATAGGATTATTTCTGATTTGCGTAGCGACAATAAGCGGTTGCGCGTCAGAGTCAAAACTACCGGAACCTCCGATGGTCAGTGTGGATTCGAGCCTGATGGTCGAGCCGAACTTgacgac                                                                           <  1:198961/301‑1 (MQ=255)`  
                                                                                                                                                                                                                                          `gatgtgaTTTGGGTTAGTATGGCATCTAGGGTCAGACTCAATGGACGCTAAATGGAAACAGGAGGT‑‑ACAATGAGTACGTTAAGAGAGTTGAGGCTGCGAAGAGCACTCAAAGAGCAATCTATGCGGTATCTGCTAAGTATCAAGAAGACCTTGCCGCGCTGGAAGGGAGCACTGATAGGATTATTTCTGATTTGCGTAGCGACAATAAGCGGGTGCGCGTCAGAGTCAAAACTACCGGAACCTCCGATGGTCAGTGTGGATTCGAGCCTGATGGTCGATCCGAAAGTGACGACCGAGATGc                                                                   <  1:245045/295‑1 (MQ=255)`  
                                                                                                                                                                                                                                              `cTATTCGGGTTAGGATGGCATCTAGGGTCAGACTCAATGGACGCTAAATGGAAACATGAGGT‑‑ACAATGAGTACGTTAAGAGAGTTGAGGCTGCGAAGAGCACTCAAAGAGCAATCGATGCGGTATCTGCTAAGTATCAAGAAGACCTTGCCGCGCTGGAAGGGAGCACTGATAGGATTATTTCTAATTTGCGTAGCGACAATAAGCGGTTGCGCGTCAGAGTCAAAACTACCTTAACCTCCGATTGTCCGTGTGTATTCGAGCCTGATGGTCGAGCCGAACTTGACGAACGCAGTGCTaaa                                                               >  1:38956/1‑301 (MQ=255)`  
                                                                                                                                                                                                                                                `gatgtgtaaaagagacagtCTAGGGTCAGACTTAATGGACGCTAAATGGAAACAGGAGGT‑‑ACAATGAGTACGTTAAGAGAGTTGAGGCTGCGAAGAGCACTCAAAGAGCAATCGATGCGGTATCTGCTAAGTATCAAGAAGACCTTGCCGCGCTGGAAGGGAGCACTGATAGGATTATTTCTGATTTGCGTAGCGACAATAAGCGGTTGCGCGTCAGAGTCAAAACTACCGGAACCTCCGATGGTCAGTGTGGATTCGAGCCTGATGGTCGAGCCGAACTTGACGACCGAGATGCTAAACg                                                             <  1:322625/283‑1 (MQ=255)`  
                                                                                                                                                                                                                                                `agtCGGGTATGGATGGAATTTAGGGTCGGATGGAAGGGAGGCTAAATGGAAACAGGAGTT‑‑ACAATGAGTACGTTAAGAGAGTTGAGGCTGCGAAGAGAACTGAAGGATCAATCGATGCGGTATCTGCTAAGTATCAATAAGACCTTGCCGCGCTGGAAGGGAGCACTGATAGGATTATTTCTGATTTGCGTAGCGACAATAAGCGGTTGCGCGTCAGAGTCAACACTACCGGCACCGCGGAGGGTCAGTGTGGATTCGAGCCTGATGGTCGAGCCGAACTTGACGACCGAGATGCTAAACg                                                             <  1:249632/299‑1 (MQ=255)`  
                                                                                                                                                                                                                                                `aTTCGGGTTAGGATGGCATCTAGGGTCAGACTCAATGGACGCTAAATGGAAACAGGAGGT‑‑ACAATGAGTACGTTAAGAGAGTTGAGGCTGCGAAGAGCACTCAAAGAGCAATCGATGCGGTATCTGCTAAGTATCAAGAAGACCTTGCCGCGCTGGAAGGGAGCACTGATAGGATTATTTCTGATTTGCGTAGCGACAATAAGCGGTTGCGCGTCAGAGTCAAAACTACCGGAACCTCCGATGGTCAGTGTGGATTCGAGCCTGATGGTCGAGCCGAACTTGACGACCGAGATGCTAAACg                                                             >  1:132983/1‑301 (MQ=255)`  
                                                                                                                                                                                                                                                           `aaagagaaagaGGGTCAGACTCAATGGACGCTAAATGGAAACAGGAGGT‑‑ACAATGAGTACGTTAAGAGAGTTGAGGCTGCGAAGAGCAATAAAAGAGCAATCGATGCGGTATCTGCTAAGTATCAAGAAGACCTTGCCGCGCTGGAAGGGAGCACTGATAGGATTATTTCTGATTTGCGTAGCGACAATAAGCGGTTGCGCGTCAGAGTCAAAACTACCGGAACCTCCGATTGTCAGTGTGGATTCGAGCCTGATGGTCGAGCCGAACTTGACGACCGAGATGCTAAACGTATTCTCGCAg                                                  <  1:440700/291‑1 (MQ=255)`  
                                                                                                                                                                                                                                                                 `agacaGGGTCAGACTCAATGGACGCTAAATGGAAACAGGAGGTACACAATGAGTACGTTAAGAGAGTTGAGGCTGCGAAGAGCACTCAAAGAGCAATCGATGCGGTATCTGCTAAGTATCAAGAAGACCTTGCCGCGCTGGAAGGGAGCACTGATAGGATTATTTCTGATTTGCGTAGCGACAATAAGCGGTTGCGCGTCAGAGTCAAAACTACCGGAACCTCCGATGGTCAGTGTGGATTCGAGCCTGATGGTCGAGCCGAACTTGACGACCGAGATGCTAAACGTATTCTCGCAGTGAc                                              <  1:168372/297‑1 (MQ=255)`  
                                                                                                                                                                                                                                                                 `aTCTAGGGTCAGACTCAATGGACGCTAAATGGAAACAGGAGGT‑‑ACAATGAGTACGTTAAGAGAGTTGAGGCTGCGAAGAGCACTCAAAGAGCAATCGATGCGGTATCTGCTAAGTATCAAGAAGACCTTGCCGCGCTGGAAGGGAGCACTGATAGGATTATTTCTGATTTGCGTAGCGACAATAAGCGGTTGCGCGTCAGAGTCAAAAATACCGGAACTTCCGATGGTCAGTGTGGATTCGAGCCTGATGGTCGAGCCGAACTTGACGACCGAGATGCTAAACGTATTCTGGCAGGGAccc                                            >  1:449498/1‑301 (MQ=255)`  
                                                                                                                                                                                                                                                                      `gggTCAGACTCAATGGACGCTAAATGGAAACAGGAGGT‑‑ACAATGAGTACGTTAAGAGAGTTGAGGCTGCGAAGAGCACTCAAAGAGCAATCGATGCGGTATCTGCTAAGTATCAAGAAGACCTTGCCGCGCTGGAAGGGAGCACTGATAGGATTATTTCTGATTTGCGTAGCGACAATAAGCGGTTGCGCGTCAGAGTCAAAACTACCGGAACCTCCGATGGTCAGTGTGGATTAGAGCCTGATGGTCGAGCCGAACTTGACGACCGAGATGCTAAACGTATTCTCGCAGTGACCCAGAAg                                       <  1:381357/301‑1 (MQ=255)`  
                                                                                                                                                                                                                                                                      `gggTCAGACTCAATGGACGCTAAATGGAAACAGGAGGT‑‑ACAATGAGTACGTTAAGAGAGTTGAGGCTGCGAAGAGCACTCAAAGAGCAATCGATGCGATATCTGCTAAGTATCAAGAAGACCTTGCCGCGCTGGAAGGGAGCACTGATAGGATTATTTCTGATTTGCGTAGCGACAATAAGCGGTTGCGCGTCAGAGTCAAAACTACCGGAACCTCCGATGGTAAGTGTGGATTCGAACCTGATGGTCGAGCCGAACTTGACGACCGAGATGCTAAACGTATTCTCGCAGTGACCCAGAAg                                       >  1:298027/1‑301 (MQ=255)`  
                                                                                                                                                                                                                                                                       `ggTCAGACTCAATGGACGCTAAATGGAAACAGGAGGTACACAATGCGTACGCTAATAGAGTTGAGGCTGCGAAGAGCACTCAAAGAGCAATCGATGCGTTATCTGCTAAGTATCAAGCAGACCTTGCCGCGCTGGAAGGGAGCACTGATAGGATTATTTCTGATTTGCGTATCGACAATAAGCGGTTGCGCGTCAGAGCCAAAACTACCGGAACCTCCGATGGCCAGTGTGGATTCGAGCCTGATGGTCGAGCCGAACTTGACGACCGAGCTGCTAAACGTATTCTCGCATTGACTCAGaa                                        >  1:360220/1‑301 (MQ=255)`  
                                                                                                                                                                                                                                                                       `aacaggACTCAAAGGACGGGAAAGGAAAACAGGAGGTAAACAAGGATTACGTTAAGAAAGTTGAGGCGGAGAAGAGAACTAAAAGAGAAATGGATGCGGTAAATGCTAAGTATCAAGAAGACCTTGCCGCGCTGGAAGGGAGCACTGATAGGATTATTTCTGATTTGCGTAGCGACAATAAGCGGTTGCGCGTCAGAGTCAAAACTACCGGAACCTCCGATGGTCAGTGTGGATTCGAGCCTGATGGTCGAGCCGAACTTGACGACCGAGATGCTAAACGTATTCTCGCAGTGACCCAGaa                                        <  1:91074/296‑1 (MQ=255)`  
                                                                                                                                                                                                                                                                        `gTCAGACTCAATGGACGCTAAATGGAAACAGGAGGTACACAATGAGTACGTTAAGAGAGTTGAGTCTGCGAAGAGCACTCAAAGAGCAATCGATGCGGTATCTGCTAAGTATCAAGGAGACCTTGCAGCGCTGGAAGGGAGCACTGATAGGATTATTTCTGATTTGCGTAGCGACAATAAGCGGTTGAGCGTCAGAGTCAAAACTACCGGAACCTCCGATGGTCAGTGTGGATTCGAGCCTGATGGTCGAGCCGAACTTGACGACCGAGATGCTAAACGTATTCTCGCAGTGACCCAGAAg                                       <  1:489254/301‑1 (MQ=255)`  
                                                                                                                                                                                                                                                                            `gACTCAATGGACGCTAAATGGAAACAGGAGGT‑‑ACAATGAGTACGTTAAGAGAGTTGAGGCTGCGAAGAGCACTCAAATAGCAATCGATGCGGTATCTGCTTAGTATCAAGAAGACCTTGCCGCGTTGGTAGGGAGCACTGATAGGATTATTTCTGATTTGCGTAGCGACAATAAGCGGTTGCGCGTCAGAGTCAAAACTACCGGAACCTCCGTTGGTCAGTGTGGATTCGAGCCTGATGGTCGAGCCGAACTTGACGACCGTGATGCTAAACGTATTCTCGCATTGACCCAGAAGGGTGAc                                 >  1:394252/1‑301 (MQ=255)`  
                                                                                                                                                                                                                                                                              `cTCAATGGACGCTAAATGGAAACAGGAGGT‑‑ACAATGAGTACGTTAAGAGAGTTGAGGCTGCGAAGAGCACTCAAAGAGCAATCGATGCGGTATCTGCTAAGTATCAAGAAGACCTTGCCGCGCTGGAAGGGAGCACTGATAGGATTATTTCTGATTTGCGTAGCGACAATAAGCGGTTGCGCGTCAGAGTCAAAACTACCGGAACCTCCGATGGTCAGTGTGGATTCGAGCCTGATGGTCGAGCCGAACTTGACGACCGAGATGCTAAACGTATTCTCGCAGTGACCCAGAAGGGTGACGc                               >  1:215044/1‑301 (MQ=255)`  
                                                                                                                                                                                                                                                                                     `gACGCTAAATGGAAACAGGAGGT‑‑ACAATGAGTACGTTAAGAGAGTTGAGGCTGCGAAGAGCACTCAAAGAGCAATCGATGCGGTATCTGCTAAGTATCAAGAAGACCTTGCCGCGCTGGAAGGGAGCACTGATAGGATTATTTCTGATTTGCGTAGCGACAATAAGCGGTTGCGCGTCAGAGTCAAAACTACCGGAACCTCCTATGGTCAGTGTGGATTCGAGCCTGATGGTCGAGCCTAACTTGACGACCGAGATGCTAAACGTATTCTCGCAGTGACCCAGAAGGGTGACGCATGGAtt                        <  1:12941/301‑1 (MQ=255)`  
                                                                                                                                                                                                                                                                                     `gACGCTAAATGGAAACAGGAGCT‑‑ACAATGAGTACGTTAAGAGAGTTGAGGCTGCGAAGAGCACTCAAAGAGAAATCGATGCGGTATCTGCTAAGTATCAAGAAGACCTTGCCGCGCTGGAAGGGAGCACTGATAGGATTATTTCTGATTTGCGTAGCGACAATAAGCGGTTGCGCGTCAGAGTCAAAACTACCGGAACCTCCGATGGTCAGTGTGGATTCGAGCCTGATGGTCGAGCCGAACTTGACGAACGAGATGCTAAACGTATTCTCGCAGTGACCCAGAAGGGTGACGCATGGAtt                        <  1:355732/301‑1 (MQ=255)`  
                                                                                                                                                                                                                                                                                       `cGCTAAATGGAAACAGGAGGT‑‑ACAATGAGTACGTTAAGAGAGTTGAGGCTGCGAAGAGCACTCAAAGAGCAATCGATGCGGTATCTGCTAAGTATCAAGAAGACCTTGCCGCGCTGGAAGGGAGCACTGATAGGATTATTTCTGATTTGCGTAGCGACAATAAGCGGTTGCGCGTCAGAGTCAAAACTACCGGAACCTCCGATGGTCAGTGTGGATTCGAGCCTGATGGTCGAGCCGAACTTGACGACCGAGATGCTAAACGTATTCTCGCAGTGACCCAGAAGGGTGACGCATGGATTCg                      >  1:85968/1‑301 (MQ=255)`  
                                                                                                                                                                                                                                                                                               `ggAAACAGGAGGT‑‑ACAATGAGTACGTTAAGAGAGTTGAGGCTGCGAAGAGCACTCAAAGAGCAATCGATGCGGTATCTGCTAAGTATCAAGAAGACCTTGCCGCGCTGGAAGGGAGCACTGATAGGATTATTTCTGATTTGCGTAGCGACAATAAGCGGTTGCGCGTCAGAGTCAAAACTACCGGAACCTCCGATGGTCAGTGTGGATTCGAGCCTGATGGTCTAGCCGAACTTGACGACCGAGATGCTAAACGTATTCTCGCAGTGACCCAGAAGGGTGACTCATGGATTCGTGCGTTAc              >  1:207562/1‑301 (MQ=255)`  
                                                                                                                                                                                                                                                                                               `ggAAACAGGAGGT‑‑ACAATGAGTACGTTAAGAGAGTTGAGGCTGCGAAGAGCACTCAAAGAGCAATCGATGCGGTATCTGCTAAGTATCAAGAAGACCTTGCCGCGCTGGAAGGGAGCACTGATAGGATTATTTCTGATTTGCGTAGCGACAATAAGCGGTTGCGCGTCAGAGTCAAAACTACCGGAACCTCCGATGGTCAGTGTGGATTCGAGCCTGATGGTCGAGCCGAACTTGACGACCGAGATGCTAAACGTATTCTCGCAGTGACCCAGAAGGGTGACGCATGGATTCGTGCGTTAc              >  1:236630/1‑301 (MQ=255)`  
                                                                                                                                                                                                                                                                                               `ggAAACAGGAGGT‑‑ACAATGAGTACGTTAAGAGAGTTGAGGCTGCGAAGAGCACTCAAAGAGCAATCGATGCGGTATCTGCTAAGTATCAAGAAGACCTTGCCGCGCTGGAAGGGAGCACTGATAGGATTATTTCTGATTCGCGTAGCGACAATAAGCGGTTGCGCGTCAGAGGCAAAACTACCGGAACCTCCGATGGTCAGTGTTGATTCGAGCCTGATGGACGAGCCGAACTTGACGCACGAGATGCTAAACGTATTCTCGAAGTGACCCAGAAGGGTGACGCATGGATTCGTGCGTTAc              >  1:282363/1‑301 (MQ=255)`  
                                                                                                                                                                                                                                                                                                       `gaggTACACAATGAGTACGTTAAGAGAGTTGAGGCTGCGAAGAGCACTCAAAGAGCAATCGATGCGGTATCTGCTAAGTATCAAGAAGACCTTGCCGCGCTGGAAGGGAGCACTGATAGGATTATTTCTGATTTGCGTAGCGACAATAAGCGGTTGCGCGTCAGAGTCAAAACTACCGGAACCTCCGATGGTCAGTGTGGATTCGAGCCTGATGGTCGAGCCGAACTTGACGACCGAGATGCTAAACGTATTCTCGCAGTGACCCAGAAGGGTGACGCATGGATTCGTGCGTTACAGGATa        >  1:444880/1‑301 (MQ=255)`  
                                                                                                                                                                                                                                                                                                        `aagTACACAATGAGTACGTTAAGAGAGTTGAGGCTGAGAAGAGCACTCAAAGAGCAATCGATGCGGTATCTGCGAAGTATCAAGAAGACCTTGCCGGGCTGGAAGGGAGCACTGATAGGATTATTTCTGAGTTGCGTAGCGACAATAAGCGGTTGCGCGTCAGAGTTAAAACTACCGGAACCTCCGATGGTCAGTGTGGATTCGAGCCTGATGGTCGAGCCGAACTTGACGACCGAGATGCTAAACGTATTCTCGCAGTGACCCAGAAGGGTGACGCATGGATTCGTGCGTTACAGGATAc       <  1:290168/299‑1 (MQ=255)`  

                                                                                                                                                                                                                                                                                                            `||`                                                                                                                                                                                                                                                                                                              
`CACATCTTAGGTGGCCTTGCTGGTGCTCTTGAAGAGTACAAAGAGAAAGTCGGTGATAACGGTCTTACGGATGATGATATTTACACATTACAGTGATATACTCAAGGCCACTACAGATAGTGGTCTTTATGGATGTCATTGTCTATACGAGATGCTCCTACGTGAAATCTGAAAGTTAACGGGAGGCATTATGCTAGAATTTTTACGTAAGCTAATCCCTTGGGTTCTCGCTGGGATGCTATTCGGGTTAGGATGGCATCTAGGGTCAGACTCAATGGACGCTAAATGGAAACAGGAGGTACACAATGAGTACGTTAAGAGAGTTGAGGCTGCGAAGAGCACTCAAAGAGCAATCGATGCGGTATCTGCTAAGTATCAAGAAGACCTTGCCGCGCTGGAAGGGAGCACTGATAGGATTATTTCTGATTTGCGTAGCGACAATAAGCGGTTGCGCGTCAGAGTCAAAACTACCGGAACCTCCGATGGTCAGTGTGGATTCGAGCCTGATGGTCGAGCCGAACTTGACGACCGAGATGCTAAACGTATTCTCGCAGTGACCCAGAAGGGTGACGCATGGATTCGTGCGTTACAGGATACTATTC  >  NC_001604.1/36727‑37328`

  

Alignment Legend

Aligned base mismatch/match (shaded by quality score): `ATCG/ATCG` `< 3 ≤` `ATCG/ATCG` `< 10 ≤` `ATCG/ATCG` `< 18 ≤` `ATCG/ATCG` `< 32 ≤` `ATCG/ATCG` `< 38 ≤` `ATCG/ATCG`

Unaligned base: `atcg`    Masked matching base: `atcg`    Alignment gap: `‑`    Deleted base: `‑`
